# National and regional prevalence rates of hypertension in Saudi Arabia: A descriptive analysis using the national survey data

**DOI:** 10.3389/fpubh.2023.1092905

**Published:** 2023-04-04

**Authors:** Aqeel M. Alenazi, Bader A. Alqahtani

**Affiliations:** ^1^Department of Health and Rehabilitation Sciences, College of Applied Medical Sciences, Prince Sattam Bin Abdulaziz University, Al-Kharj, Saudi Arabia; ^2^Department of Health and Rehabilitation Sciences, Prince Sattam Bin Abdulaziz University, Al-Kharj, Saudi Arabia

**Keywords:** high blood pressure (HBP), epidemiology, hypertension, Saudi, prevalence

## Abstract

**Background:**

Limited studies have examined the prevalence of hypertension (HTN) at the national and regional levels in Saudi Arabia. Therefore, this study aimed to examine the national and regional prevalence of HTN in the Saudi population.

**Methods:**

This study used the data from household health survey carried out by the General Authority for Statistics in 2017. It included 24,012 households representing the Saudi population across all 13 administrative regions. The diagnosis of HTN was confirmed by a self-reported history of a physician diagnosed HTN.

**Results:**

The prevalence of HTN was 9.2% among Saudi population aged 15 years and older. It was relatively higher in women (10.0%) than in men (8.5%). The prevalence of HTN increased with advancing age (aged 65 years and older), accounting for 55.3% in women and 48.0% in men; its prevalence was relatively low among the younger age group, accounting for 0.1% in those aged 15–19 years. A large difference was found in the prevalence of HTN between regions, ranging from 6.0% in Najran region to 10.0% in Makkah region.

**Conclusion:**

This study reported the national and regional prevalence of HTN among Saudi adults using a representative sample with large variations in the prevalence rates according to age, sex, and regions. Older age, men, and Makkah region had higher prevalence of HTN. Our findings will help determine the etiological factors, identify the priorities for healthcare, and generate initiatives for policymakers, and develop preventive and therapeutic strategies for HTN.

## Introduction

Hypertension (HTN) is a common modifiable disease affecting the health of the global population and leading to morbidity and mortality (1). The presence of HTN increases the risk of several health conditions such as cardiovascular diseases, metabolic diseases, and kidney diseases (2). HTN was not only associated with cardiovascular diseases, but also with other musculoskeletal diseases such as osteoarthritis (3–5). The prevalence of HTN differs depending on the region, country, and the study sample. Thus, HTN has a significant impact on the global economy owing to its high direct and indirect costs (6). The burden of HTN in individuals and healthcare systems is relatively high due to the increasing need for health consultations, hospitalization, laboratory and radiographic examinations, and high usage of prescribed and non-prescribed medications (7). The global financial burden of HTN was estimated to be 10% of the healthcare expenditures (8). Therefore, the recent guideline has updated the criteria for diagnosing HTN: 130 mm Hg or greater for the systolic pressure and/or 80 mm Hg or greater for the diastolic pressure (9).

The prevalence of HTN is expected to increase regionally and globally due to urbanization, physical inactivity, and unhealthy diet (8). The prevalence of HTN differs in eastern and western countries. The estimated prevalence of HTN varied based on regions: 45.4% in the United States (10), 18%−44.7% in China (11, 12), and 23% in Canada (13). In addition to the discrepancy in the prevalence of HTN in western and eastern countries, this finding was also observed in the HTN prevalence in the Middle East countries including Saudi Arabia. Previous studies reported the estimated HTN prevalence rates of 4.9% in the Saudi population (14) and 15.2% in the general population (15). The prevalence has increased in the past decade from 8.9% in some regions (16), to 15.2% in other regions (15). This increase might be linked to several demographical and economic factors.

Although some large-scale studies have examined the prevalence of HTN in the national and regional levels in Saudi Arabia, these studies may have certain limitations (15, 16). Only two large-scale studies examining the prevalence of HTN were conducted, one at a regional level in 1989–1994 (16), and one at a national level (15). Al-Nozha reported that the highest prevalence of HTN was in Farasan (Jazan region) (8.9%) while the lowest prevalence of HTN was in Asir region (2.2) (16). In this study, females had higher prevalence of HTN compared to males in all regions except Altaif and Asir regions (16). However, this study had some limitations such as not reporting the overall prevalence based on gender across the country and the included data were in 1998 that could be different than current population. Another study by El Bcheraoui et al. reported that the prevalence of HTN was 15.2% with no specification to regional prevalence (15). This study reported that males had higher prevalence of HTN across all age groups except 55–64 years group (48.3% for males vs. 48.6 for females) (15). None of those studies have reported the regional and national prevalence rates of HTN according to age, sex, and regions all together. Hence, insufficient evidence about HTN in Saudi Arabia was 10 years ago and more. Therefore, it is necessary to update the prevalence information of HTN in Saudi Arabia. Considering the growing prevalence of HTN, data from previous studies may differ from the current estimates of HTN. Hence, necessary strategies to examine the prevalence of HTN in Saudi Arabia are warranted to determine the etiological factors, identify the priorities for healthcare, generate initiatives for policymakers, and develop preventive and therapeutic strategies for HTN. Therefore, it is crucial to comprehensively examine the HTN prevalence in Saudi Arabia. Thus, this study aimed to examine the prevalence of HTN in the Saudi population according to age groups, sex, and regions using the national level data in Saudi Arabia.

## Materials and methods

As part of a comprehensive Kingdom-wide screening, the General Authority for Statistics (GASTAT) carried out a continuous household health survey. A total of 24,012 households that are representative of the survey population and evenly distributed across the Kingdom's administrative areas served as the basis for selecting the survey sample. This study was approved by the University Research Ethics Committee of Prince Sattam Bin Abdulaziz University (RHPT/021/017). Due to the retrospective nature of the study and use of published reports, the requirement for obtaining informed consent was waived.

A pilot survey was conducted between October and November of 2017 to identify issues during data collection and make necessary modifications before the actual start of data collection. Therefore, actual data collection was between November and December of 2017 using interviewing method. All data were collected using soft copies *via* a software with filed researchers to ensure quality of data. Data auditing started afterward of data collection and a second data auditing was conducted in May 2018.

The selection of sample units from the statistical structures that included the target population was performed in two stages. In the first stage, the primary sampling units were determined. The enumeration regions, which were a component of the enumeration and coding phases of buildings and residential properties, were chosen as sampling units.

**Figure 1:**
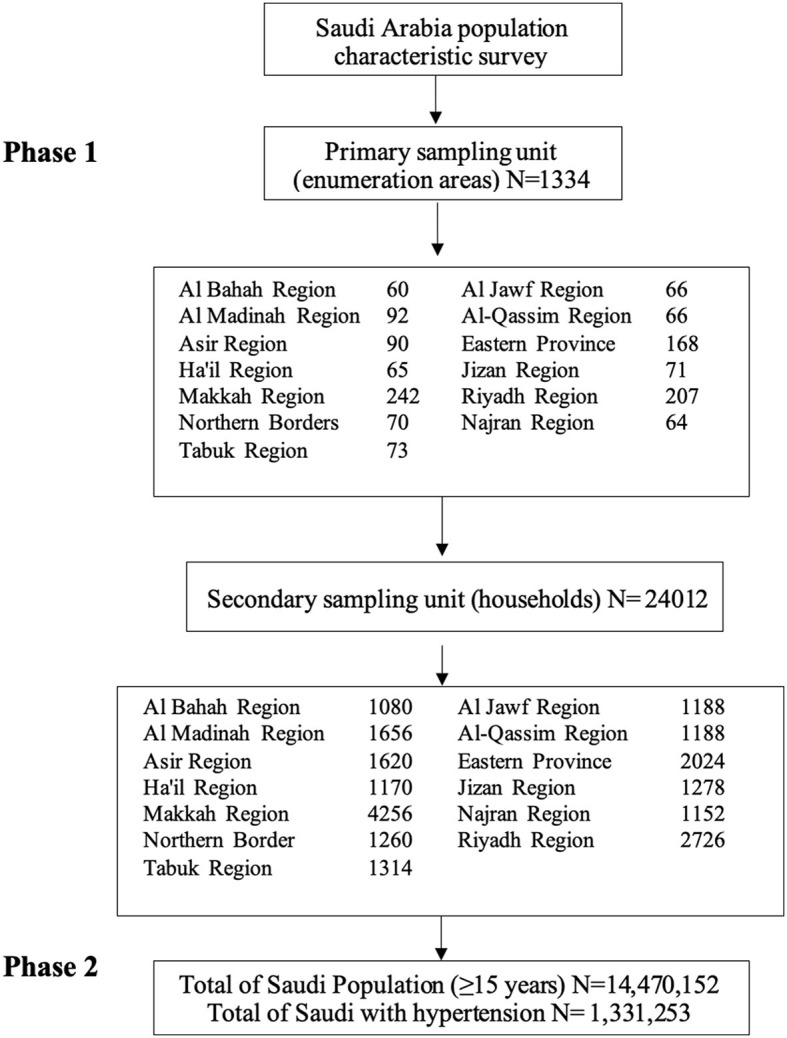
The flowchart of the survey sample selection.

A total of 1,334 enumeration areas were selected from the administrative regions by using a proportional-size method and weighing the total number of Saudi households. The final sampling units were selected at random from the statistical areas in the second phase. In this phase, the households identified in the first phase's enumeration zones had been chosen through regular random sampling, yielding a total of 24,012 households (Figure 1).

A trained field researcher from the GASTAT interviewed the head of each household and recorded all information on an iPad. The members of the household personally filled out the survey questionnaire and provided the data. The field researchers went to the houses of the survey participants and tracked their location using their tablets based on the map coordinates. Upon reaching the participants' home, the field researchers introduced themselves and showed their official identification cards. They also explained the purpose of their visit and provided an outline of the survey and its goals. The electronic questionnaire was personally completed by the head of the household or an adult member of the family who was familiar with the participant's health status. Only patients with a confirmed diagnosis of HTN who had undergone the required testing and was informed of their diagnosis by a specialist doctor were included in the study.

### Sample size

The process of selecting the Primary Sampling Units (PSUs) from the primary sample framework was initiated after establishing the ideal home survey sample size for each administrative region based on a systematic population that is proportionate to the target size (number of eligible households) from a sorted list. The main sample calculated from the counting areas was 1,334. The samples were allocated to different study groups based on the established categories in every location (Figure 1).

### Statistical analysis

The data were analyzed using the Stata software version 15.1 (Stata Corp, College Station, TX). A thematic mapping of the prevalence of HTN was generated using a web-based map customization tool (SimpleMaps.com, Pareto Software, LLC, USA). In the current study, the prevalence rates (%) of HTN (i.e., the basic descriptive epidemiology) in the entire sample were calculated. The prevalence of HTN was also calculated for the age-specific, gender-specific, and administrative region-stratified subsamples. Odds ratio (OR) for gender and presence or absence of HTN was calculated along with 95% Confidence Interval (95%CI) for the OR and prevalence rate.

## Results

**Figure 2:**
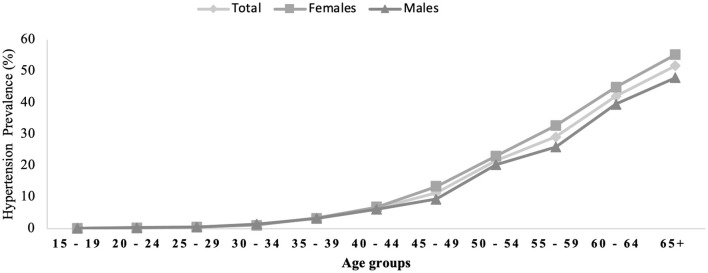
Hypertension prevalence across gender and age groups.

The overall prevalence of HTN was 9.2% [95% CI: (9.20, 9.22%)] among Saudi adults aged 15 years and older. Women showed higher prevalence of HTN (10.0%) [95% CI: (9.98, 10.02%)] than men (8.5%) [95% CI: (8.48, 8.52%)]. Figure 2 illustrates the proportion of Saudi individuals aged 15 years and older who were diagnosed with HTN according to gender and age. The results showed that the females had greater odds of having HTN when compared to males [OR: 1.074; 95% CI (1.071, 1.078), *p* < 0.001].

The prevalence of Saudi individuals diagnosed with HTN dramatically increased with age; the prevalence increased at the age of 40 years and peaked at the age of 65 years and older. It increased in adults aged 65 years and older (women: 55.3%; men: 48.0%). Meanwhile, the lowest prevalence was found among the 15–19-year age group (0.1%).

**Figure 3:**
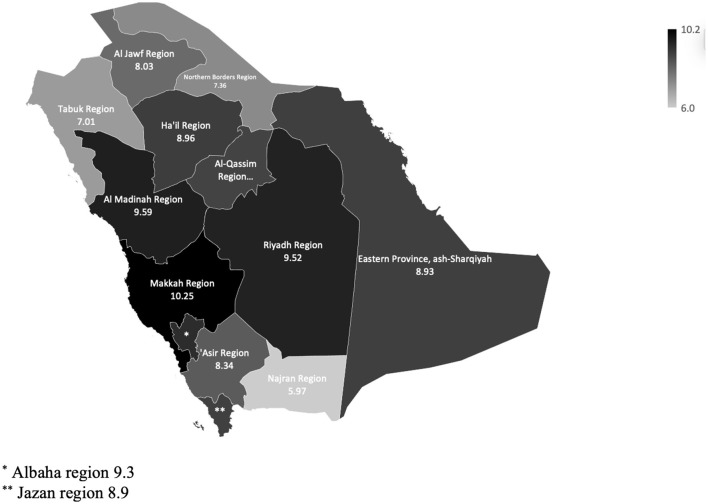
The national and regional prevalence rates of hypertension.

Figure 3 shows the national and prevalence rates of HTN across the 13 administrative regions in Saudi Arabia. The Makkah Al-Mukarramah region had the highest prevalence of HTN (10.0%), followed by Al Madinah (9.6%). Meanwhile, the Najran region had the lowest prevalence of HTN (6.0%).

## Discussion

The current study reported the prevalence of HTN among Saudi individuals aged 15 years and older using a national representative sample. This study reported an overall HTN prevalence of 9.2% across all regions in Saudi Arabia (men: 8.5%; women: 10.0%). Large differences were observed in the prevalence of HTN according to age, sex, and region. This study showed that adults aged 65 years and older (51.7%), women, and those living in the Makkah region (10.0%) had the highest prevalence of HTN. The lowest prevalence of HTN was reported among younger adults aged 15–19 years (0.1%), men, and those living in the Najran region (6.0%). This study was the first to report the prevalence of HTN at the national level representing all administrative regions of Saudi Arabia.

Saudi Arabia is one of the largest countries in the Middle East, which showed rapid economic growth in the past decades. These economic changes across the country led to the socioeconomic growth among individuals and served as a barrier to the risk reduction of chronic diseases such as HTN. In addition, environmental barriers such as climate is another challenge among the Saudi population. In Saudi Arabia, the temperature frequently rises as high as 55°C in the summer, sandstorms occur, and the average annual rainfall is only <5 inches (report, 2023). Hence, the population has become highly dependent on vehicles to decreased physical activity. The presence of domestic helpers such as drivers and housekeepers may lead to physical inactivity and uncontrolled diet. Individuals in Saudi Arabia prefer social gathering that may limit diet control and time to perform exercise (17). These lifestyle changes, urbanization, and socioeconomic status possibly contributed to the increased risk of HTN among individuals living in the Saudi Arabian regions (18). These factors could affect lifestyle and behavior including poor diet, and level of physical activity, thus increasing the risk of diseases (19, 20). Recently, the prevalence of numerous diseases has increased in Saudi Arabia including diabetes, HTN, stroke incidence, osteoarthritis, and frailty (5, 14, 15, 20–22).

Studies evaluating the prevalence of HTN among Saudi adults are limited. Studies from Saudi Arabia reported a lower prevalence of HTN (4.9–7.1%) compared with that in our study (15, 23). Aldiab et al. also reported a lower prevalence of HTN (4.9%) compared with that in our study (9.2%), which included individuals aged 18–67 years (14). This inconsistency might be related to the region (Alkharj city) and the included sample (*n* = 1,019) (14). Another possible factors contributing to the low prevalence of HTN in Alkharj city were its semi-urban population and environmental characteristics (e.g., climate) (14). Another large-scale study at the national level reported that the prevalence of self-reported HTN in 2013 was 7.1% (15). Moreover, the prevalence of HTN reported in our study was relatively high (9.2%) compared with that reported in El Bcheraoui et al.'s study (7.1%) (15). In the same study, the participants' blood pressure was measured, and results showed an HTN prevalence of 15.2% (15). This finding highlights the importance of undergoing screening to identify individuals with undiagnosed HTN as this significantly impacts public health.

A few population-based studies have been conducted to examine the prevalence of HTN in Saudi Arabia. The results of these studies were in contrast with our findings since they reported higher prevalence of HTN in the Saudi population (15, 24, 25). Bcheraoui et al. reported that 15.2% of the included participants had HTN, which was either measured or self-reported (15). Another previous study reported that the prevalence of HTN was 25.5% among adults (24). Al-Nozha et al. reported an HTN prevalence of 26.1% among 17,230 participants (25). Although previous studies used larger samples, some factors possibly contributed to the discrepancy in our findings. The age groups and inclusion criteria differed between studies. In previous studies, the data were collected before 2013; in the current study, data collection was conducted in 2018. Previous studies included individuals who underwent blood pressure measurement to assess for HTN rather than those with self-reported HTN. Therefore, future studies should examine the risk factors associated with HTN, especially the modifiable factors.

Very few studies reported the prevalence of HTN according to age and gender similar to our groups. El Bcheraoui et al. reported somehow similar categories for age groups according to gender (15). Our results showed lower prevalence of HTN when compared to El Bcheraoui et al.'s study. For example, in older adults aged 65 years and older, our study found that the prevalnce of HTN among males and females was 48.0 and 55.3%, respectively compared to El Bcheraoui et al.'s study that reported 68.1 and 61% among males and females, respectively (15). Other age groups were different in categories including 10 years compared to 5 years intervals in our study (15). Another study reported the prevalnce of HTN among older males (44.6%) and females (50.4%), respectively compared to our study (48.0 and 55.3%, respectively) (25). However, a difference should be noted in the category as this study included adults aged 60–70 years in one category while our study included adults aged 65 years and older in tis age category.

The overall prevalence of HTN (9.2%) reported in the present study was substantially lower than the prevalence reported worldwide and in other countries. The present study reported the global HTN prevalence rates of 32% among women and 34% among men aged 30–79 years (26). Another study showed a global HTN prevalence of 31.1% (8). According to the country-level data, the prevalence of HTN was estimated to be 30.5% in Korea (27). Previous studies conducted in the United States showed an estimated HTN prevalence of 32%, while that conducted in China reported an estimated HTN prevalence of 23.2% (26). Although the previously mentioned prevalence of HTN was higher worldwide and in some other countries, the results could be attributed to methodological differences such as age and diagnosis. For example, previous large-scale studies included individuals aged 30 years and older who had a systolic blood pressure of 140 mm Hg or greater, a diastolic blood pressure of 90 mm Hg or greater, or were taking antihypertensive drugs (26, 27). In our study, we included individuals aged 15 years and older with self-reported HTN, which could possibly explain the low prevalence of HTN in our population. Hence, future studies should examine the prevalence of HTN using objective measures based on the updated guidelines for HTN diagnosis.

Some limitations should be considered when interpretating our findings. Although a self-reported HTN questionnaire has acceptable validity and reliability in the literature, it failed to identify those who had undiagnosed HTN in our study (28, 29). Further studies are needed to examine those with HTN and to determine the validity of results using gold standard diagnostic tools and measures in the Saudi population. The limited information related to this condition is another limitation of this study as the results did not specifically indicate the HTN stage (1 or 2). We did not measure other risk factors related to the occurrence of HTN such as obesity, poor diet, and lack of physical activity. Hence, future studies should investigate HTN in terms of prevalence and the modifiable factors among the Saudi population at the national level.

## Conclusion

This study reported the national and regional prevalence of HTN among Saudi adults using a representative sample with large variations in prevalence according to age, sex, and region. Older age, men, and those living in the Makkah region had higher prevalence of HTN. Future research should use high-quality studies that used gold standard measures to establish preventive strategies for HTN in Saudi Arabia. The findings of the current study will help determine the etiological factors, identify the priorities for healthcare, generate initiatives for policymakers, and develop preventive and therapeutic strategies for HTN.

## Data availability statement

Publicly available datasets were analyzed in this study. This data can be found here: https://www.stats.gov.sa/en.

## Ethics statement

Ethical review and approval was not required for the study on human participants in accordance with the local legislation and institutional requirements. Written informed consent for participation was not required for this study in accordance with the national legislation and the institutional requirements.

## Author contributions

Both authors contributed equally to the study conception and design. Material preparation, data collection, and analysis were performed by both authors. The first draft of the manuscript was written by both authors and both authors commented on previous versions of the manuscript. Both authors read and approved the final manuscript.
